# Bis(cyanato-κ*N*)tetra­kis­(2,6-dimethyl­pyrazine-κ*N* ^4^)nickel(II)

**DOI:** 10.1107/S1600536812027985

**Published:** 2012-06-23

**Authors:** Susanne Wöhlert, Inke Jess, Christian Näther

**Affiliations:** a Institut für Anorganische Chemie, Christian-Albrechts-Universität Kiel, Max-Eyth-Strasse 2, 24118 Kiel, Germany

## Abstract

Reaction of nickel(II) chloride with sodium cyanate and 2,6-di­methyl­pyrazine leads to single crystals of the title com­pound, [Ni(NCO)_2_(C_6_H_8_N_2_)_4_]. The nickel(II) cation is located about a centre of inversion and is octa­hedrally coordinated by two cyanate anions and four 2,6-dimethyl­pyrazine ligands, forming discrete complexes.

## Related literature

For the background to this work relating to complexes with thio­cyanato and seleno­cyanato and *N*-donor ligands, see: Boeckmann & Näther (2010 ▶); Wriedt *et al.* (2009 ▶); Boeckmann *et al.* (2010 ▶).
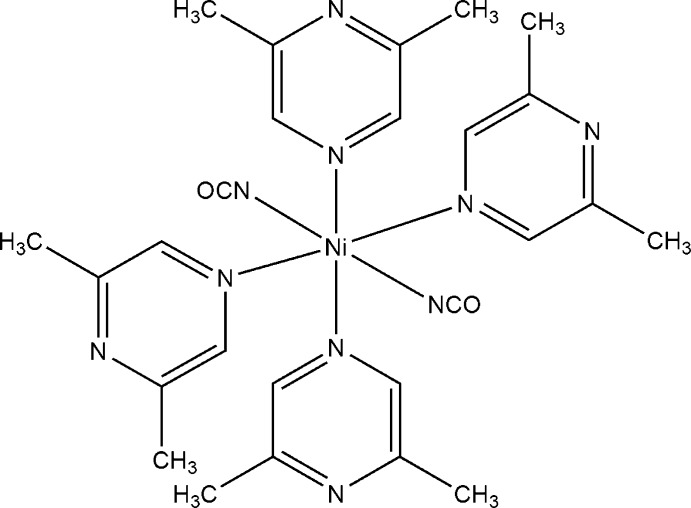


## Experimental

### 

#### Crystal data

[Ni(NCO)_2_(C_6_H_8_N_2_)_4_]
*M* *_r_* = 575.33
Monoclinic, 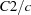

*a* = 24.932 (2) Å
*b* = 8.4963 (3) Å
*c* = 18.1748 (13) Å
β = 133.148 (7)°
*V* = 2808.9 (3) Å^3^
*Z* = 4
Mo *K*α radiation
μ = 0.73 mm^−1^
*T* = 293 K
0.07 × 0.04 × 0.03 mm

#### Data collection

Stoe IPDS-2 diffractometer
Absorption correction: numerical (*X-SHAPE* and *X-RED32*; Stoe & Cie 2008 ▶) *T*_min_ = 0.888, *T*_max_ = 0.969
8293 measured reflections
3341 independent reflections
2516 reflections with *I* > 2σ(*I*)
*R*_int_ = 0.047

#### Refinement

*R*[*F* ^2^ > 2σ(*F* ^2^)] = 0.039
*wR*(*F* ^2^) = 0.102
*S* = 1.01
3341 reflections
182 parameters
H-atom parameters constrained
Δρ_max_ = 0.30 e Å^−3^
Δρ_min_ = −0.40 e Å^−3^

### 

Data collection: *X-AREA* (Stoe & Cie, 2008 ▶); cell refinement: *X-AREA*; data reduction: *X-AREA*; program(s) used to solve structure: *SHELXS97* (Sheldrick, 2008 ▶); program(s) used to refine structure: *SHELXL97* (Sheldrick, 2008 ▶); molecular graphics: *XP* in *SHELXTL* (Sheldrick, 2008 ▶) and *DIAMOND* (Brandenburg, 2011 ▶); software used to prepare material for publication: *XCIF* in *SHELXTL*.

## Supplementary Material

Crystal structure: contains datablock(s) I, global. DOI: 10.1107/S1600536812027985/zl2488sup1.cif

Structure factors: contains datablock(s) I. DOI: 10.1107/S1600536812027985/zl2488Isup2.hkl

Additional supplementary materials:  crystallographic information; 3D view; checkCIF report

## Figures and Tables

**Table 1:** Selected bond lengths (Å)

Ni1—N1	2.0396 (19)
Ni1—N10	2.1475 (17)
Ni1—N20	2.1983 (15)

**Fig. 1.**
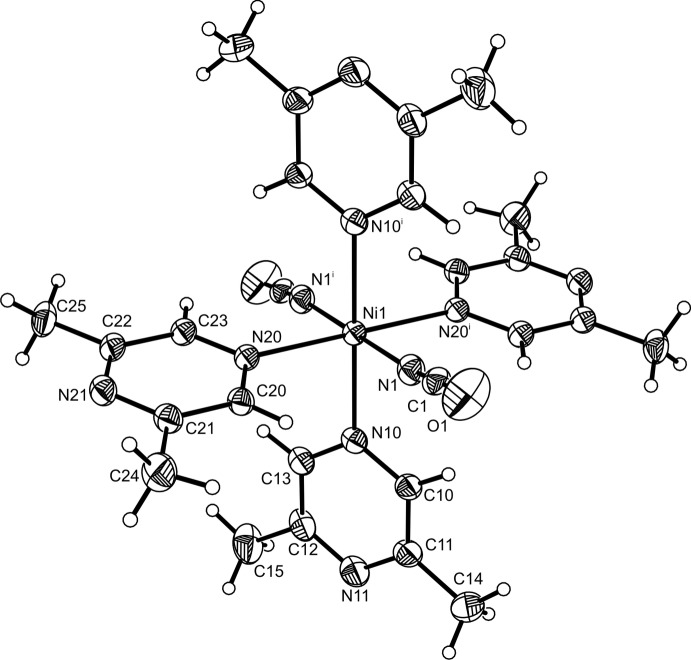
: Crystal structure of the title compound with labeling and displacement ellipsoids drawn at the 50 % probability level. Symmetry code: i = -x + 3/2, -y + 3/2, -z + 1.

## supplementary crystallographic information

### Comment

Recently we have reported on the synthesis, structures and properties of new coordination polymers based on paramagnetic transition metals, small-sized anionic ligands such as thiocyanato and selenocyanato, and *N*-donor ligands. In the course of these investigations we found that new coordination compounds with bridging anionic ligands can be prepared by thermal decomposition of suitable precursor compounds, in which the anionic ligands are only terminally coordinated (Wriedt *et al.*, 2009 and Boeckmann & Näther, 2010). In further investigations we also have shown that even metal formate coordination compounds can be prepared by this method (Boeckmann, Wriedt & Näther, 2010). In our current investigations we tried to prepare similar compounds based on transition metal cyanates with 2,6-dimethylpyrazine as a neutral co-ligand. Therefore, we have reacted nickel(II) chloride with sodium cyanate and 2,6-dimethylpyrazine which resulted in the formation of crystals of the title compound that were identified by single crystal X-ray diffraction.

The asymmetric unit of the title compound consists of one nickel(II) cation, which is located on a centre of inversion, one cyanato anion and two 2,6-dimethylpyrazine ligands in general positions (Fig. 1). In the crystal structure each nickel(II) cation is coordinated by two terminal N-bonded cyanato anions and four 2,6-dimethylpyrazine ligands into discrete complexes, and the coordination polyhedra around the Ni cations corresponds to a slightly distorted octahedra. The anionic ligands are trans to each other and because of sterical crowding the 2,6-dimethylpyrazine ligand is coordinated via the nitrogen atom that is not neighbouring the methyl groups. The Ni—N distances range from 2.0396 (19) Å to 2.1983 (15) Å with angles between 88.72 (6) ° and 180 ° (Table 1). The shortest intermolecular distances between the nickel(II) cations is 8.4963 (3) Å.

### Experimental

Nickel(II) chloride hexahydrate (NiCl_2_×6H_2_O), sodium cyanate (NaNCO) and 2,6-dimethylpyrazine were obtained from Alfa Aesar. All chemicals were used without further purification. 0.15 mmol (35.5 mg) NiCl_2_×6H_2_O and 0.3 mmol (19.5 mg) NaNCO were reacted in 0.6 mmol (65 µL) 2,6-dimethylpyrazine. Green shaped single-crystals suitable for structure determination were obtained after one week at room temperature.

### Refinement

C—H H atoms were positioned with idealized geometry (methyl H atoms were allowed to rotate but not to tip) and were refined isotropically with *U*_iso_(H) = 1.2*U*_eq_(C) (1.5 for methyl H atoms) and C—H distances of 0.93 Å for aromatic and 0.96 Å for methyl H atoms using a riding model.

### Crystal data

**Table:**

[Ni(NCO)_2_(C_6_H_8_N_2_)_4_]	*F*(000) = 1208
*M**_r_* = 575.33	*D*_x_ = 1.360 Mg m^−^^3^
Monoclinic, *C*2/*c*	Mo *K*α radiation, λ = 0.71073 Å
Hall symbol: -C 2yc	Cell parameters from 8293 reflections
*a* = 24.932 (2) Å	θ = 2.7–28.0°
*b* = 8.4963 (3) Å	µ = 0.73 mm^−^^1^
*c* = 18.1748 (13) Å	*T* = 293 K
β = 133.148 (7)°	Block, green
*V* = 2808.9 (3) Å^3^	0.07 × 0.04 × 0.03 mm
*Z* = 4	

### Data collection

**Table:**

Stoe IPDS-2 diffractometer	3341 independent reflections
Radiation source: fine-focus sealed tube	2516 reflections with *I* > 2σ(*I*)
Graphite monochromator	*R*_int_ = 0.047
ω scan	θ_max_ = 28.0°, θ_min_ = 2.7°
Absorption correction: numerical (*X-SHAPE* and *X-RED32*; Stoe & Cie 2008)	*h* = −25→32
*T*_min_ = 0.888, *T*_max_ = 0.969	*k* = −11→10
8293 measured reflections	*l* = −23→21

### Refinement

**Table:**

Refinement on *F*^2^	Primary atom site location: structure-invariant direct methods
Least-squares matrix: full	Secondary atom site location: difference Fourier map
*R*[*F*^2^ > 2σ(*F*^2^)] = 0.039	Hydrogen site location: inferred from neighbouring sites
*wR*(*F*^2^) = 0.102	H-atom parameters constrained
*S* = 1.01	*w* = 1/[σ^2^(*F*_o_^2^) + (0.0632*P*)^2^] where *P* = (*F*_o_^2^ + 2*F*_c_^2^)/3
3341 reflections	(Δ/σ)_max_ < 0.001
182 parameters	Δρ_max_ = 0.30 e Å^−^^3^
0 restraints	Δρ_min_ = −0.40 e Å^−^^3^

### Special details

**Table:**

Geometry. All esds (except the esd in the dihedral angle between two l.s. planes) are estimated using the full covariance matrix. The cell esds are taken into account individually in the estimation of esds in distances, angles and torsion angles; correlations between esds in cell parameters are only used when they are defined by crystal symmetry. An approximate (isotropic) treatment of cell esds is used for estimating esds involving l.s. planes.
Refinement. Refinement of F^2^ against ALL reflections. The weighted R-factor wR and goodness of fit S are based on F^2^, conventional R-factors R are based on F, with F set to zero for negative F^2^. The threshold expression of F^2^ > 2sigma(F^2^) is used only for calculating R-factors(gt) etc. and is not relevant to the choice of reflections for refinement. R-factors based on F^2^ are statistically about twice as large as those based on F, and R- factors based on ALL data will be even larger.

### Fractional atomic coordinates and isotropic or equivalent isotropic displacement parameters (Å^2^)

**Table:**

	*x*	*y*	*z*	*U*_iso_*/*U*_eq_	
Ni1	0.7500	0.7500	0.5000	0.01887 (11)	
N20	0.64456 (8)	0.8103 (2)	0.34913 (11)	0.0212 (3)	
N10	0.79329 (8)	0.6983 (2)	0.43337 (11)	0.0231 (4)	
C1	0.68822 (10)	0.4093 (3)	0.46580 (14)	0.0255 (4)	
N1	0.71677 (9)	0.5219 (2)	0.47971 (13)	0.0275 (4)	
N21	0.50923 (9)	0.8818 (2)	0.15700 (13)	0.0270 (4)	
C10	0.82299 (10)	0.5593 (3)	0.44444 (15)	0.0261 (4)	
H10	0.8239	0.4804	0.4807	0.031*	
C23	0.62225 (10)	0.9586 (2)	0.31901 (15)	0.0237 (4)	
H23	0.6526	1.0401	0.3631	0.028*	
N11	0.85370 (10)	0.6399 (3)	0.35157 (14)	0.0364 (5)	
C22	0.55452 (10)	0.9952 (3)	0.22309 (15)	0.0251 (4)	
C20	0.59907 (10)	0.6966 (3)	0.28228 (14)	0.0239 (4)	
H20	0.6130	0.5918	0.3002	0.029*	
O1	0.65623 (11)	0.2844 (2)	0.45025 (18)	0.0568 (6)	
C11	0.85283 (11)	0.5294 (3)	0.40292 (15)	0.0312 (5)	
C13	0.79369 (10)	0.8085 (3)	0.38110 (14)	0.0267 (4)	
H13	0.7725	0.9060	0.3708	0.032*	
C12	0.82493 (11)	0.7812 (3)	0.34183 (16)	0.0330 (5)	
C14	0.88376 (15)	0.3709 (3)	0.4124 (2)	0.0454 (6)	
H14A	0.8580	0.3295	0.3464	0.068*	
H14B	0.8780	0.3006	0.4479	0.068*	
H14C	0.9350	0.3812	0.4490	0.068*	
C15	0.82941 (15)	0.9083 (4)	0.2884 (2)	0.0526 (8)	
H15A	0.8799	0.9357	0.3276	0.079*	
H15B	0.8029	0.9994	0.2800	0.079*	
H15C	0.8082	0.8707	0.2234	0.079*	
C21	0.53149 (10)	0.7326 (3)	0.18668 (14)	0.0272 (4)	
C25	0.53126 (11)	1.1627 (3)	0.19022 (17)	0.0341 (5)	
H25A	0.5382	1.1915	0.1461	0.051*	
H25B	0.5604	1.2302	0.2483	0.051*	
H25C	0.4803	1.1738	0.1552	0.051*	
C24	0.48127 (12)	0.6054 (3)	0.11260 (18)	0.0405 (6)	
H24A	0.4342	0.6160	0.0917	0.061*	
H24B	0.5019	0.5045	0.1437	0.061*	
H24C	0.4756	0.6143	0.0549	0.061*	

### Atomic displacement parameters (Å^2^)

**Table:**

	*U*^11^	*U*^22^	*U*^33^	*U*^12^	*U*^13^	*U*^23^
Ni1	0.01411 (16)	0.02221 (18)	0.01902 (17)	−0.00050 (14)	0.01083 (13)	0.00029 (14)
N20	0.0161 (7)	0.0272 (8)	0.0213 (7)	0.0021 (6)	0.0131 (6)	0.0022 (6)
N10	0.0158 (7)	0.0312 (9)	0.0196 (7)	−0.0012 (6)	0.0111 (6)	−0.0006 (6)
C1	0.0202 (9)	0.0288 (11)	0.0239 (9)	0.0043 (8)	0.0137 (8)	−0.0008 (8)
N1	0.0210 (8)	0.0326 (10)	0.0254 (8)	0.0028 (7)	0.0145 (7)	0.0032 (7)
N21	0.0194 (7)	0.0364 (10)	0.0244 (8)	0.0039 (7)	0.0147 (7)	0.0044 (7)
C10	0.0234 (9)	0.0324 (11)	0.0243 (9)	0.0031 (8)	0.0171 (8)	0.0017 (8)
C23	0.0181 (8)	0.0290 (10)	0.0244 (9)	0.0022 (7)	0.0146 (8)	0.0021 (8)
N11	0.0289 (9)	0.0556 (13)	0.0335 (9)	0.0103 (8)	0.0248 (8)	0.0102 (9)
C22	0.0179 (8)	0.0344 (11)	0.0270 (9)	0.0061 (8)	0.0169 (8)	0.0062 (8)
C20	0.0180 (8)	0.0299 (10)	0.0200 (9)	0.0013 (7)	0.0116 (8)	0.0001 (7)
O1	0.0535 (11)	0.0307 (10)	0.0848 (15)	−0.0174 (8)	0.0467 (12)	−0.0082 (9)
C11	0.0245 (10)	0.0445 (13)	0.0267 (10)	0.0061 (9)	0.0183 (9)	0.0020 (9)
C13	0.0204 (9)	0.0361 (11)	0.0240 (9)	0.0012 (8)	0.0153 (8)	0.0037 (8)
C12	0.0230 (9)	0.0498 (15)	0.0281 (10)	0.0058 (8)	0.0183 (8)	0.0114 (9)
C14	0.0521 (14)	0.0546 (16)	0.0491 (14)	0.0189 (12)	0.0422 (13)	0.0102 (12)
C15	0.0500 (14)	0.0681 (19)	0.0591 (17)	0.0181 (14)	0.0449 (14)	0.0293 (15)
C21	0.0192 (8)	0.0378 (13)	0.0218 (9)	0.0020 (8)	0.0129 (8)	0.0004 (8)
C25	0.0263 (10)	0.0380 (13)	0.0365 (11)	0.0115 (9)	0.0210 (9)	0.0139 (9)
C24	0.0270 (10)	0.0422 (14)	0.0292 (10)	−0.0009 (10)	0.0102 (9)	−0.0066 (10)

### Geometric parameters (Å, º)

**Table:**

Ni1—N1^i^	2.0396 (19)	C22—C25	1.498 (3)
Ni1—N1	2.0396 (19)	C20—C21	1.395 (3)
Ni1—N10	2.1475 (17)	C20—H20	0.9300
Ni1—N10^i^	2.1475 (17)	C11—C14	1.502 (3)
Ni1—N20	2.1983 (15)	C13—C12	1.389 (3)
Ni1—N20^i^	2.1983 (15)	C13—H13	0.9300
N20—C23	1.335 (3)	C12—C15	1.507 (3)
N20—C20	1.345 (3)	C14—H14A	0.9600
N10—C10	1.334 (3)	C14—H14B	0.9600
N10—C13	1.339 (3)	C14—H14C	0.9600
C1—N1	1.113 (3)	C15—H15A	0.9600
C1—O1	1.238 (3)	C15—H15B	0.9600
N21—C22	1.339 (3)	C15—H15C	0.9600
N21—C21	1.341 (3)	C21—C24	1.497 (3)
C10—C11	1.398 (3)	C25—H25A	0.9600
C10—H10	0.9300	C25—H25B	0.9600
C23—C22	1.400 (3)	C25—H25C	0.9600
C23—H23	0.9300	C24—H24A	0.9600
N11—C11	1.334 (3)	C24—H24B	0.9600
N11—C12	1.347 (3)	C24—H24C	0.9600
			
N1^i^—Ni1—N1	180.00 (13)	N11—C11—C10	121.2 (2)
N1^i^—Ni1—N10	89.86 (7)	N11—C11—C14	117.3 (2)
N1—Ni1—N10	90.14 (7)	C10—C11—C14	121.5 (2)
N1^i^—Ni1—N10^i^	90.14 (7)	N10—C13—C12	121.8 (2)
N1—Ni1—N10^i^	89.86 (7)	N10—C13—H13	119.1
N10—Ni1—N10^i^	180.000	C12—C13—H13	119.1
N1^i^—Ni1—N20	89.74 (7)	N11—C12—C13	121.1 (2)
N1—Ni1—N20	90.26 (7)	N11—C12—C15	117.1 (2)
N10—Ni1—N20	88.72 (6)	C13—C12—C15	121.7 (2)
N10^i^—Ni1—N20	91.28 (6)	C11—C14—H14A	109.5
N1^i^—Ni1—N20^i^	90.26 (7)	C11—C14—H14B	109.5
N1—Ni1—N20^i^	89.74 (7)	H14A—C14—H14B	109.5
N10—Ni1—N20^i^	91.28 (6)	C11—C14—H14C	109.5
N10^i^—Ni1—N20^i^	88.72 (6)	H14A—C14—H14C	109.5
N20—Ni1—N20^i^	180.000	H14B—C14—H14C	109.5
C23—N20—C20	116.73 (16)	C12—C15—H15A	109.5
C23—N20—Ni1	122.62 (13)	C12—C15—H15B	109.5
C20—N20—Ni1	120.64 (14)	H15A—C15—H15B	109.5
C10—N10—C13	116.95 (19)	C12—C15—H15C	109.5
C10—N10—Ni1	122.49 (14)	H15A—C15—H15C	109.5
C13—N10—Ni1	120.51 (15)	H15B—C15—H15C	109.5
N1—C1—O1	179.7 (2)	N21—C21—C20	121.5 (2)
C1—N1—Ni1	167.16 (17)	N21—C21—C24	117.32 (18)
C22—N21—C21	117.19 (17)	C20—C21—C24	121.1 (2)
N10—C10—C11	121.7 (2)	C22—C25—H25A	109.5
N10—C10—H10	119.2	C22—C25—H25B	109.5
C11—C10—H10	119.2	H25A—C25—H25B	109.5
N20—C23—C22	122.01 (19)	C22—C25—H25C	109.5
N20—C23—H23	119.0	H25A—C25—H25C	109.5
C22—C23—H23	119.0	H25B—C25—H25C	109.5
C11—N11—C12	117.2 (2)	C21—C24—H24A	109.5
N21—C22—C23	121.1 (2)	C21—C24—H24B	109.5
N21—C22—C25	117.86 (17)	H24A—C24—H24B	109.5
C23—C22—C25	121.1 (2)	C21—C24—H24C	109.5
N20—C20—C21	121.5 (2)	H24A—C24—H24C	109.5
N20—C20—H20	119.3	H24B—C24—H24C	109.5
C21—C20—H20	119.3		

Symmetry code: (i) −*x*+3/2, −*y*+3/2, −*z*+1.
